# Flexible multivariate hemodynamics fMRI data analyses and simulations with PyHRF

**DOI:** 10.3389/fnins.2014.00067

**Published:** 2014-04-10

**Authors:** Thomas Vincent, Solveig Badillo, Laurent Risser, Lotfi Chaari, Christine Bakhous, Florence Forbes, Philippe Ciuciu

**Affiliations:** ^1^INRIA, MISTIS, LJK, Grenoble UniversityGrenoble, France; ^2^UNATI/INRIA Saclay, Parietal, CEA/DSV/I^2^BM NeuroSpin centerGif-sur-Yvette, France; ^3^INRIA, Parietal, NeuroSpin centerGif-sur-Yvette, France; ^4^CNRS, UMR 5219, Statistics and Probability Team, Toulouse Mathematics InstituteToulouse, France; ^5^INP-ENSEEIHT/CNRS UMR 5505, TCI, IRIT, University of ToulouseToulouse, France

**Keywords:** medical imaging analysis, fMRI, Bayesian inference, python, scientific computing

## Abstract

As part of fMRI data analysis, the pyhrf package provides a set of tools for addressing the two main issues involved in intra-subject fMRI data analysis: (1) the localization of cerebral regions that elicit evoked activity and (2) the estimation of activation dynamics also known as Hemodynamic Response Function (HRF) recovery. To tackle these two problems, pyhrf implements the Joint Detection-Estimation framework (JDE) which recovers parcel-level HRFs and embeds an adaptive spatio-temporal regularization scheme of activation maps. With respect to the sole detection issue (1), the classical voxelwise GLM procedure is also available through nipy, whereas Finite Impulse Response (FIR) and temporally regularized FIR models are concerned with HRF estimation (2) and are specifically implemented in pyhrf. Several parcellation tools are also integrated such as spatial and functional clustering. Parcellations may be used for spatial averaging prior to FIR/RFIR analysis or to specify the spatial support of the HRF estimates in the JDE approach. These analysis procedures can be applied either to volume-based data sets or to data projected onto the cortical surface. For validation purpose, this package is shipped with artificial and real fMRI data sets, which are used in this paper to compare the outcome of the different available approaches. The artificial fMRI data generator is also described to illustrate how to simulate different activation configurations, HRF shapes or nuisance components. To cope with the high computational needs for inference, pyhrf handles distributing computing by exploiting cluster units as well as multi-core machines. Finally, a dedicated viewer is presented, which handles *n*-dimensional images and provides suitable features to explore whole brain hemodynamics (time series, maps, ROI mask overlay).

## 1. Introduction

As Magnetic Resonance Imaging (MRI) is a growing imaging modality in neuroscience, the need for powerful tools to explore the increasing amount of data is more and more significant. This data growth is quantitative as cohort sizes are getting bigger through the development of international multi-center projects like the Human Brain Project (Kandel et al., 2013) but also qualitative as high field magnets become more and more available (Duyn and Koretsky, 2011). Functional MRI (fMRI), in particular, benefits from these improvements. The experimenter has access to finer spatial (~1 mm) and temporal (~1 s) resolutions and also higher signal-to-noise ratio (SNR). In particular, the higher temporal resolution combined with higher SNR allows for a better recovery of dynamic processes so that we are no longer limited to only static mappings of cerebral activity. In this context, pyhrf aims to extract dynamic features from fMRI data, especially the Blood Oxygenated Level Dependent (BOLD) modality (Ogawa et al., 1990). The observed BOLD signal is an indirect measure of the neural activity via the oxygen variation induced by the neuro-vascular coupling. Therefore, analysis methods have to formalize a hemodynamic model in order to make inference on neural processes. However, even if BOLD variations are known to correlate with neural activity, it is difficult to disentangle the dynamics of neural and the vascular components. As the employed methodology mainly uses linear systems, dynamic processes are summarized within the so-called Hemodynamic Response Function (HRF), which is the impulse response that links neuronal activity to the fMRI signal. In fact, the package offers various tools to analyze evoked fMRI data ranging from spatial mappings such as those provided by the General Linear Model (GLM) framework (Friston et al., 1995) to finer hemodynamics models as provided by the joint detection-estimation (JDE) approach described in Makni et al. (2005, 2008) and Vincent et al. (2010). Through a bilinear and time-invariant system, the JDE approach models an unknown HRF at the level of a group of voxels (referred to as a parcel in the following) as well as voxel- and condition-specific response levels to encode the local magnitudes of this response. The HRF is only constrained to be smooth (temporal regularization) and can cover a wide variety of shapes. The response levels are spatially regularized within each parcel. Hence, the JDE approach is a spatially adaptive GLM built on unknown parcel-dependent HRFs with spatio-temporal regularization.

The use of each tool depends on a choice of model which is driven by the features required by the experimenter's goal. To obtain classical detection results, a GLM based on the canonical HRF (and possibly its temporal derivatives) may be sufficient. Even if the between-region hemodynamic variability is acknowledged, the canonical HRF can provide good results in regions where it has been calibrated such as temporal and occipital cortices as studied by Boynton et al. (1996). However, in regions involving more complex processes, HRF derivatives or function bases may not be enough to capture the hemodynamic fluctuations allowing activation detection. Hemodynamics delays may happen due to varying reaction delays or pathological cases. Moreover, if one is interested in studying the dynamic features of the response, an *explicit* HRF estimation is required. The main question in this case concerns whether there is a need for condition-specific features or not. That is, a need for an HRF estimation associated with each experimental condition or for a single HRF estimate associated with all conditions. If explicit condition-wise HRFs are required, the best methodological tool to use is the temporally Regularized FIR (RFIR) developed in Ciuciu et al. (2003) and Marrelec et al. (2003). Otherwise, if variability is only expected across separated and specialized regions, the JDE framework is well-suited. Indeed, within a specialized region, if only one condition exhibits activity then the region-specific HRF can be considered a condition-specific HRF. The performance of RFIR models depends nonetheless on the number of experimental conditions involved in the paradigm because the higher this number, the larger the number of parameters to estimate, and thus the fewer the number of degrees of freedom for statistical testing. The model choice thus also depends on the experimental paradigm. First, it is worth noticing that the use of the JDE formulation is less relevant in the analysis of block paradigm data since the signal variability in this case is small. The JDE formalism is actually more adapted to fast event-related paradigms or to paradigms including many conditions, like the localizer paradigm (10 conditions) introduced by Pinel et al. (2007) and used hereafter in this paper. The JDE approach is also optimally tuned to combined analysis of hemodynamics features with the detection of activated brain areas. To summarize, the JDE model choice provides a fair compromise with the possibility for the user to adapt the model to the studied region.

JDE also delivers interesting and complementary results for the sole activation-detection aspect compared with classical GLM. Spatial regularization, which is necessary due to the low SNR in fMRI, is not enforced in the same way between methods. In the GLM, FIR, and RFIR cases, there is no embedded spatial regularization within models. Indeed, the data are usually spatially smoothed with a fixed Gaussian kernel in preprocessing. In contrast, JDE incorporates spatial correlation through hidden Markov models. The amount of spatial correlation is automatically tuned and also adaptive across brain regions, therefore avoiding any prior invariant smoothing.

pyhrf is mainly written in python with some C code to cope with computationally demanding parts of algorithms. This python choice has been made possible thanks to the nipy^1^ project and especially nibabel^2^ to handle data reading/writing in the NIFTI format.

In terms of package maturity, pyhrf is a research tool which has the ambition to target cognitive neuroscientists and clinicians. Efforts are made in terms of user-friendliness and the design is a trade-off between *mutability* which is required by methodological research where specifications change frequently and *usability* where user interfaces should be as stable as possible to ease external non-developer use cases.

The rest of the paper is organized as follows. First, methods available in the package are presented, comprising parcellation and detection/estimation analyses. Then, the workflow and design of the pyhrf package are detailed. These cover the user interface and code snippets for the main analysis treatments, simulation framework, distributed computations and data viewer. Results illustrate the outcome of geometric and functional parcellations and their impact on detection/estimation treatments. Finally, conclusions are drawn and perspectives for future developments are indicated.

## 2. Methods

The are two main kinds of fMRI data analysis methods available in pyhrf: (1) parcellation tools that segment the brain into disjoint sets of positions and (2) activation detection/HRF estimation tools that highlight correlations between the input experimental paradigm and variations in the measured fMRI signal. The first kind comprises two spatial parcellation tools: Voronoi-based random parcellation, as reviewed by Aurenhammer and Klein (2000) and balanced partitioning, developed in Elor and Bruckstein (2009). The second kind comprises the GLM introduced in Friston (1998), the FIR model described in Henson et al. (2000), the RFIR model developed in Ciuciu et al. (2003) and the JDE approach presented in Vincent et al. (2010) and Risser et al. (2011). The GLM and FIR GLM procedures are provided by nipy while RFIR and JDE are originally implemented in pyhrf. For all these methods, we refer to their respective bibliographical references for an extensive presentation of their methodology. Nonetheless, the main aspects of these methods are summarized in what follows to allow comparison between them, especially in terms of model structure and assumptions.

After detailing notations, we introduce detection/estimation methods, namely GLM, FIR and RFIR, which require the measured fMRI signal and the timing of the experimental paradigm as input. After setting the generative model common to all detection/estimation methods and a brief comparative overview, each approach is presented in more details. Subsequently, parcellation methods are presented. Spatial parcellation approaches can be applied directly to the input fMRI data and only depend on its geometry. Functional parcellation, which is a clustering of GLM results, is detailed afterwards.

### 2.1. Notation

#### 2.1.1. Conventions

We denote vectors with bold lower case (e.g., 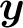) and matrices with bold upper case letters (e.g., ***P***). A vector is by convention a column vector. Scalars are denoted with non-bold lower case letters (e.g., *a*). The transpose operation is denoted by ^t^. Probability distribution functions (pdf) are denoted using calligraphic letters (e.g., 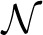 and 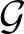 for the Gaussian and gamma distributions).

#### 2.1.2. Data geometry

As methods can be applied to data defined in the volume or on the cortical surface, the generic term “position” will be used in place of “voxel” (volume unit) and “node” (surface mesh unit). Position indexes are denoted by *j* = 1 : *J* to indicate a range between 1 and *J*. Data is assumed to be masked to only allow positions within the brain. *J* is the total number of positions within the functional mask. In addition, when considering parcellated data, this functional mask is divided into a set of Γ parcels denoted ℙ = {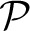
_1_, …,
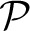
_γ_, …, 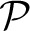
_Γ_}, where 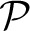
_γ_ is the set of *J*_γ_ = |
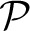
_γ_| position indexes belonging to parcel γ.

#### 2.1.3. Functional data

We consider the usual case of evoked fMRI data analysis where the experimental paradigm comprising *M* conditions is known. The signal measured at each time of repetition (*TR*) is denoted 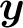
_*j*_ = {*y*_*j*,*n*_}_*n* = 1:*N*_ where *N* is the number of scans. Stimulus timing onsets for a given experimental condition *m* = 1 : *M* are encoded by variable 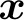
^*m*^ so that *x*^*m*^_*t*_ = 1 if a stimulus occurs at time *t* up to a time step Δ*t*, else *x*^*m*^_*t*_ = 0. The time step is such that Δ*t* ⩽ *TR* and depends on the actual temporal resolution sought by the analysis method.

### 2.2. Detection/estimation methods

For ease of comparison, the presentation of all methods is immersed in the same formalism where the signal is assumed generated by a linear and time-invariant (convolution) system with additive noise. We also consider the usual case of taking into account a position-specific low frequency drift in the data which is a well known fMRI artifact produced by the aliasing of respiratory and cardiac rhythms into the low frequencies as studied in Yan et al. (2009). The generic forward model, reads:

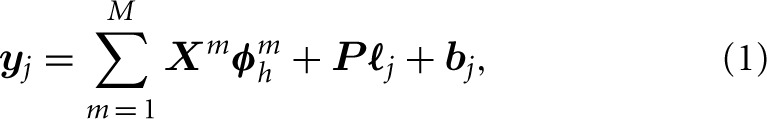



where:

- ***P*** is a fixed orthonormal basis that takes a potential drift and any other nuisance effect (e.g., motion parameters) into account. The low-frequency drift can classically be either polynomial with an order up to 5 or cosine with a cut-off of 0.01 Hz,
- ℓ_*j*_ are the unknown regression weights associated with ***P***,
- ***b***_*j*_ is the noise component,
- ***ϕ***^*m*^_*h*_ is a “generic” hemodynamic filter of size *D*. For a typical duration of 25 s, *D* = 25/TR for the GLM and FIR GLM^3^ approaches, while *D* = 25/(*TR*/4) for the RFIR and JDE approaches considering a typical oversampling factor of 4. In the GLM framework, ***ϕ***^*m*^_*h*_ can be fixed to the canonical HRF or parametric when resorting to function bases and we will note *R* the number of unknown parameters. In non-parametric approaches, all HRF coefficients are estimated as in RFIR or JDE approaches,
- ***X***^*m*^ is the *N* × *D* stimulus occurrence matrix consisting of the lagged stimulus covariates for the experimental condition *m*: ***X***^*m*^ = [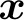
^*m*^_*t*_1__, …, 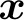
^*m*^_*t*_*N*__]^t^ with 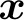
^*m*^_*t*_*n*__ = (*x*^*m*^_*t*_*n*_ − *d*Δ*t*_)^t^_0⩽*d*⩽*D*_,
- $\sum {}_{m=1}^{M}$ ***X***^*m*^ ***ϕ***^*m*^_*h*_ is hence the summation of all stimulus-induced signal components which are generated as the convolution between the paradigm encoded in ***X***^*m*^ and the hemodynamic filters ***ϕ***^*m*^_*h*_.

For the sake of simplicity, multiple-run data are not considered here but all implemented methods can handle such data with a fixed-effect model (same effect size across runs), a homoscedastic noise model (one noise variance for all runs) and run-specific drift coefficients (see discussion for further extensions).

To give a first overview of how this generative model structure is derived in the different approaches, Table 1 provides a comparison in terms of regularization, number of unknowns and analysis duration. Embedded spatial regularization is only available in the JDE procedure, while temporal regularization is available in RFIR and JDE (Table 1—1st, 2nd rows). In terms of constraints applied to the HRF shape (Table 1—3rd row), the basis set GLM (BS GLM) is the most constraining and the shape captured depends on the choice of the function basis. In the FIR, RFIR and JDE cases, any form of HRF shape can be recovered, provided that they are smooth in the case of RFIR and JDE. On Table 1—4th row, the information on the number of unknowns conveys the level of parsimony of a given model. BS GLM, FIR and RFIR have increasing model complexity as the number of parameters for the HRF increases. In contrast, JDE achieves larger parsimony by making the number of unknowns associated with the HRF dependent on the number of parcels rather than on the number of positions. When computing the ratio between the number of unknowns and the number of data points for a typical fMRI experiment (Table 1—5th row), it appears that JDE is comparable to a GLM with derivatives. The RFIR presents the worst situation with three times more unknowns than data points. In terms of analysis duration (Table 1—last row), GLM methods are almost instantaneous as their inference is straightforward. RFIR relies on an iterative scheme to perform unsupervised estimation of the amount of temporal regularization and is hence much slower. In addition, the implementation of RFIR is done in pure python with a main loop over positions which worsen its slow computation speed (~30 h. for a whole brain analysis)^4^. Therefore, this approach is rather limited to the processing of some regions of interest where we expect cerebral activity instead of whole brain data analysis. The computation speed of JDE is also slow, but to a lesser extent as results can be obtained overnight (~8 h. for a whole brain analysis) on a single processing unit. All these considerations on speed have to be nuanced with the access to increasing computing power and distributed computations, as will be seen in section 3.3.

**Table 1:** **Comparative overview for all detection/estimation analysis procedures available in pyhrf in terms of model structure and analysis duration**.

	**BS GLM**	**FIR GLM**	**RFIR**	**JDE**
Spatial regularization	Smoothing	Smoothing	Smoothing	Adaptive
Temporal regularization	None	None	2nd order deriv.	2nd order deriv.
HRF shape constraint	Function basis	Free	Smooth	Smooth
Number of unknowns for the stimulus-induced component	*J* × *R* × *M*	*J* × *D* × *M*	*J* × *M* × (*D* + 1)	2 × *J* × *M* + Γ × (*D* + 4*M* + 1)
	1 ⩽ *R* ⩽ 3	*D* ≈ 10	10 ⩽ *D* ⩽ 50	10 ⩽ *D* ⩽ 50, Γ ≈ 400
Typical ratio of unknowns/data	0.23	0.78	3.4	0.16
Analysis duration	3 min	5 min	30 h	8 h

*“2nd order deriv.” stands for a penalization on the energy of the HRF which penalizes abrupt shape changes. The number of nuisance parameters was considered the same for all models, so that only the modeling of the stimulus-induced component is relevant to assess model parsimony. The ratio “unknowns/data” is given for a typical fMRI data analysis with *J* = *4* × *10*^*4*^, *R* = *3, D* = *40, M* = *10*, Γ = *400*, and *N* = *128* (total number of data points: N × J). The analysis duration is for a whole brain data treatment on an Intel Core i5 (M480 2.67 Ghz)*.

#### 2.2.1. Basis set general linear model

**Figure 1:**
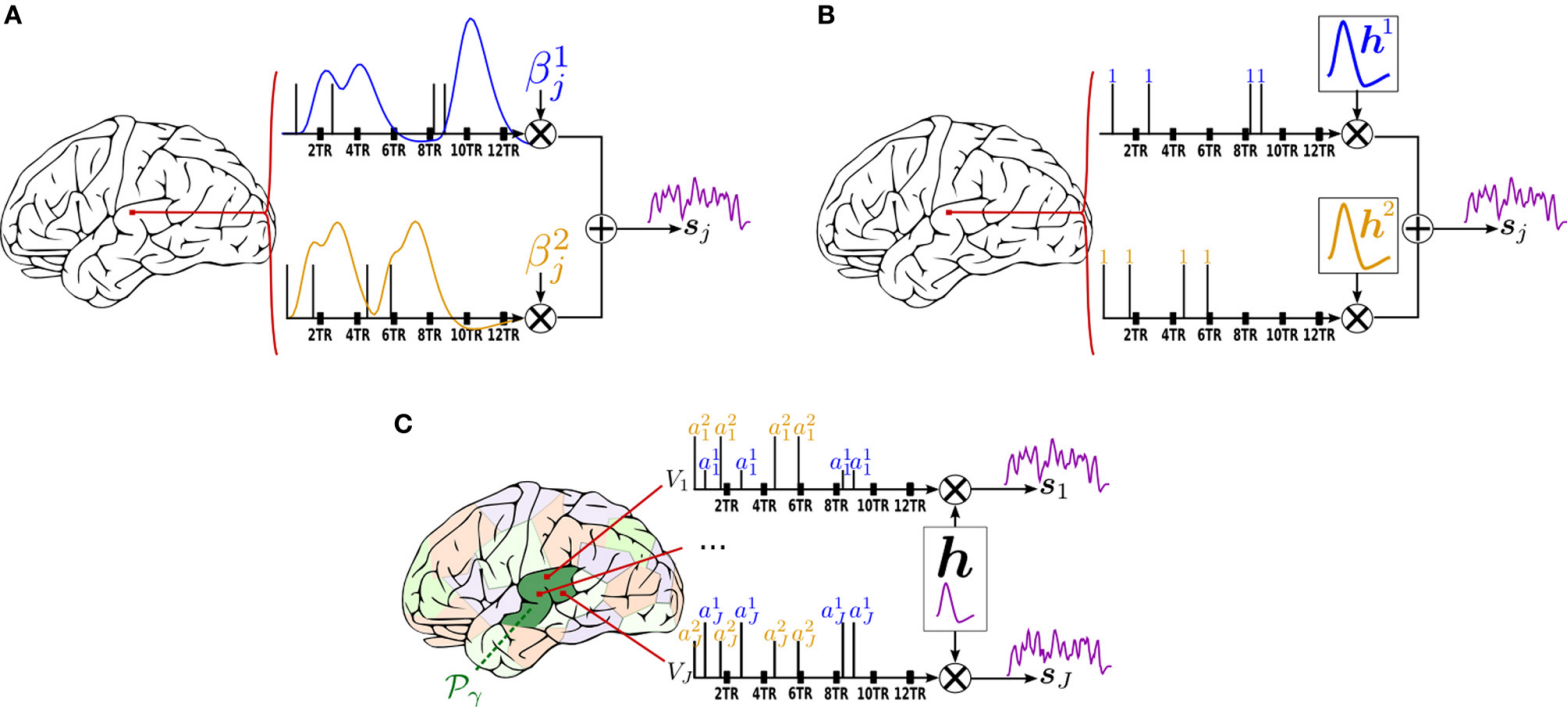
**Forward models generating the stimulus-induced components for the methods available in pyhrf**. In all cases, the scheme involves two experimental conditions colored in 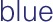 and 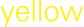 with four stimulation events as depicted by vertical bars over the TR-sampled grid. **(A)** General Linear Model (GLM). For a given condition in a given voxel, the stimulus event sequence is convolved with the fixed canonical HRF resulting in a fixed stimulus-induced regressor. This regressor is then multiplied by an unknown effect β^*m*^_*j*_. All the condition-specific regressors are then summed to form the final stimulus-induced signal *s*_*j*_. **(B)** Finite Impulse Response (FIR) Model. In a given voxel, the stimulus event-sequence is convolved with an unknown FIR vector ***h***^*m*^ for each condition to yield a condition-specific component. All components are then summed to form the final stimulus-induced signal *s*_*j*_. **(C)** Joint Detection-Estimation (JDE). For a given voxel in a given parcel 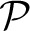
_γ_, the stimulus sequence gathering all experimental conditions is multiplied by the response levels {*a*^*m*^_*j*_}. Then, this spike signal is convolved with an unknown spatially-invariant HRF ***h*** to form the stimulus-induced signal *s*_*j*_.

In any position *j* of the brain, the basis set GLM (BS GLM) allows for some limited hemodynamic fluctuations by modeling the hemodynamic filter function ***ϕ***_*h*_ in Equation (1) as a weighted sum of the fixed canonical HRF denoted ***h***_*c*_ and its first and second order derivative ***h***′_*c*_, ***h***″_*c*_ as proposed in Friston (1998). The generative model, illustrated in Figure 1A, reads:

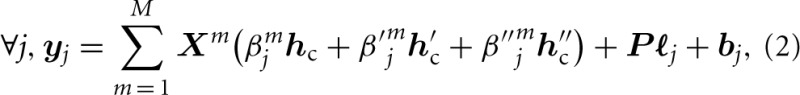



where β^*m*^_*j*_, β′^*m*^_*j*_, β″^*m*^_*j*_ are the unknown effects associated with the *m*^th^ stimulus-induced regressors constructed with the fixed known vectors ***h***_*c*_, ***h***′_*c*_, ***h***″_*c*_, respectively. To obtain the classical GLM with only the canonical HRF, β′_*j*_ and β″_*j*_ can be set to zero for all positions. It is worth noting that this formulation of the forward model is equivalent to the classical one where all regressors are gathered in the design matrix (noted ***X***) and all corresponding effects gathered in a single vector β. Equation (2) reads:

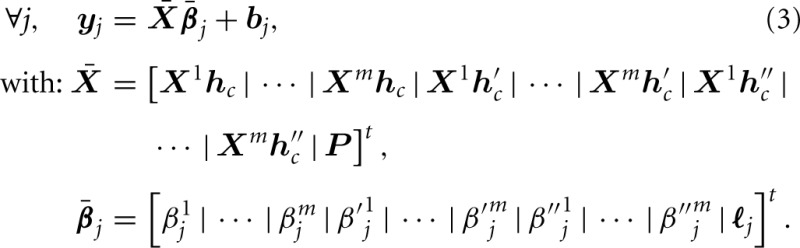



The hemodynamics fluctuations caught by such a model are limited to ~1 s around the peak of the canonical HRF which is at 5 s, see Calhoun et al. (2004). This model is massively univariate since every position *j* is analyzed independently, i.e., no correlation between neighboring signals is considered. It works well on spatially smoothed data to counter-balance the low signal-to-noise ratio, at the expense of blurred activation clusters. In the nipy implementation of the GLM, the fitting process can be performed using ordinary least squares in the case of white Gaussian noise or using Kalman filtering in the case of an *AR*(1) Gaussian noise process.

#### 2.2.2. FIR GLM and regularized FIR

The generative BOLD signal modeling in the FIR context encodes all HRF coefficients as unknown variables (illustrated in Figure 1B):

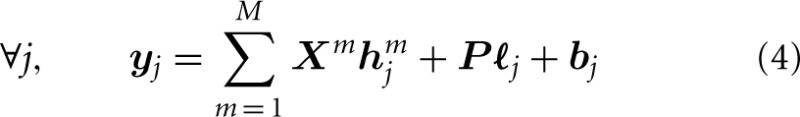



Here, vector ***h***^*m*^_*j*_ = (*h*^*m*^_*j*,*d*Δ*t*_)^t^_*d*=0,..,*D*_ represents the unknown HRF time course in voxel *j* which is associated with the *m*^th^ experimental condition and sampled every Δ*t*. In its un-regularized version, the FIR model can be expressed in the GLM framework and hence its implementation in pyhrf relies on nipy.

In the case of the Regularized FIR (Ciuciu et al., 2003), the problem is placed in the Bayesian formalism in order to inject regularity on the recovered HRF coefficients ***h***_*j*_. More specifically, ***h***^*m*^_*j*_ ~ 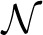
(**0**, *v*_***h***^*m*^_*j*__***R***) with ***R*** = (***D***^t^_2_***D***_2_)^−1^ where ***D***_2_ is the second-order finite difference matrix enforcing local smoothness by penalizing abrupt changes quadratically and *v*_***h***^*m*^_*j*__ is the unknown HRF prior variance which is jointly estimated. Computational and inference details are given in Ciuciu et al. (2003).

#### 2.2.3. Joint detection-estimation

The functional mask of a given subject's brain is *a priori* divided in Γ functionally homogeneous *parcels* using methods described in section 2.3.2. In each parcel 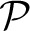
γ, the shape of the HRF ***h***_γ_ is assumed constant and the parcel-specific generative model reads:

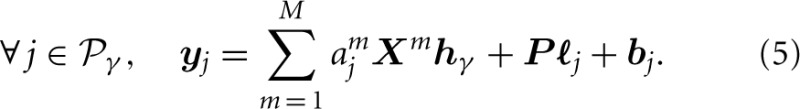



where 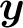
_*j*_, ***X***^*m*^, ***P***, ℓ_*j*_, and ***b***_*j*_ match the variables introduced in section 2.2.1. As shown in Figure 1C which illustrates this forward model, the *a*^*m*^_*j*_ variables encode fluctuations that occur *before* the application of the hemodynamic filter. Therefore, they are attributed to neural effects and referred to as “Neural Response Levels” (NRL). However, this term, which is historical, might be misleading as it is difficult to disentangle the contribution of the neural and the vascular components from single BOLD fMRI data. These variables can be more simply identified to the voxel- and condition-specific response amplitudes.

In contrast to Equation (2) for the GLM forward model, the fixed HRF components ***h***_*c*_ and ***h***′_*c*_ are replaced by an *unknown* parcel-based HRF ***h***_γ_. Similarly, each unknown NRL *a*^*m*^_*j*_ embodies a single magnitude parameter per regressor whereas the GLM formulation implies that the magnitude is distributed between weights β^*m*^_*j*_, β′^*m*^_*j*_, and β″^*m*^_*j*_. To summarize, the HRF shape and the BOLD response magnitude are coupled in the GLM formulation whereas they are decoupled in the JDE formulation.

In the Bayesian framework, priors are formulated to (1) enforce temporal smoothness on the HRF shape to perform estimation in the same manner as for RFIR and (2) account for spatial correlations between NRLs through spatial mixture models to perform detection, as described in Vincent et al. (2010). The spatial regularization factor is jointly estimated and optimized wrt parcel topology so as to perform an adaptive spatial smoothing. If the experimenter is not interested in the estimation of the HRF, then the HRF can be fixed typically to its canonical version in the JDE framework, which hence amounts to a *spatially adaptive GLM*. The latter approach enables parcelwise multivariate detection of activations with adaptive regularization across parcels. As shown at the group-level in Badillo et al. (2013b), this strategy retrieves more peaked and less extended activation clusters compared to classical SPM-like analysis.

The inference is performed by a stochastic sampling scheme where posterior mean estimates are computed from Markov Chain Monte Carlo samples. The implementation of the main sampling loop is coded in pure python and some intensive samplers such as the one for the HRF of the NRLs are coded in C. Still, the overall JDE procedure is computationally demanding. However, since there are as many *independent* models as parcels, the analysis can be split up into parcel-wise *parallel* analyses (see section 3.3). The efficiency of the inference scheme has also been improved by resorting to a variational formulation of the JDE (Chaari et al., 2013) which is also available in pyhrf.

### 2.3. Parcellation

#### 2.3.1. Spatial parcellation

**Figure 2:**
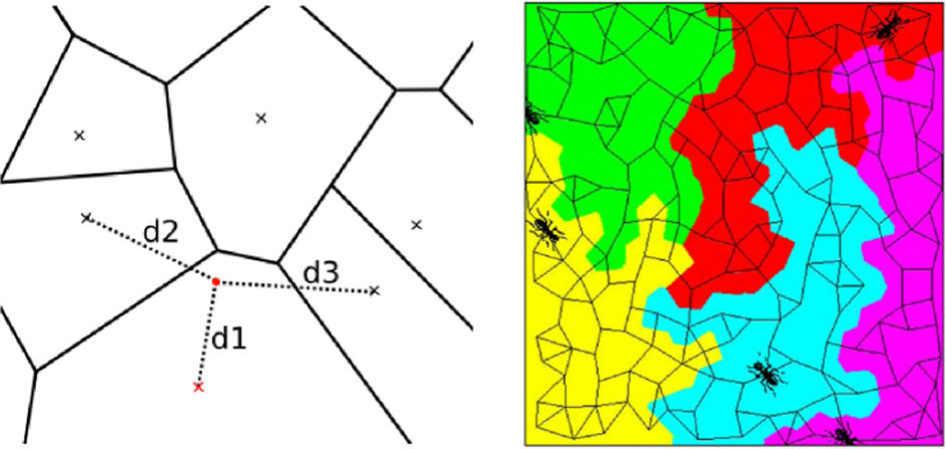
**Illustration of spatial parcellation methods in pyhrf**. **Left:** Voronoi diagram where seeds are represented as crosses. The red point is assigned to the red seed and verifies that its distance to any other seed is larger (d1 < d2, d1 < d3). **Right:** Balanced partitioning performed by patrolling a(ge)nts, image extracted from Elor and Bruckstein (2009).

***2.3.1.1. Random Voronoi diagrams***. A Voronoi diagram consists of a spatial partitioning that builds parcels around predefined control points or seeds. The parcel boundaries are placed so that each point of a given parcel is closer to the associated parcel seed than any other seed in terms of the Euclidean distance, as illustrated in Figure 2 (left). To build a parcellation from such partitioning, i.e., to assign each cerebral position to a parcel identifier, we do not explicitly require the parcel boundaries. Accordingly, there is no need to rely on classical algorithms that precisely compute these boundaries. Instead, a given position is assigned to the closest seed by resorting to a kd-tree (^5^).

Random Voronoi parcellations are convenient ways to generate samples in the space of sensible parcellations as they produce convex and compact parcels which are physiologically plausible. They have been used in Vincent et al. (2008) to study the sensitivity of the parcel-based JDE method.

***2.3.1.2. Balanced partitioning***. The goal of balanced partitioning is to build parcels of equal sizes. In the case of a non-regular topology such as the brain, there is no morphological tool to deterministically solve such partitioning problem which is known to be NP-complete as mentioned in Andreev and Räcke (2004). Hence, the algorithm implemented in pyhrf employs a heuristic and relies on a multi-agent system that mimics the inflation of balloons in a fixed volume (Elor and Bruckstein, 2009), as illustrated in Figure 2 (right).

Balanced partitioning is useful to test the effect of parcel size. In pyhrf, balanced partitioning is implemented in pure python with a position-wise main loop and is hence rather slow: ~1 min to split 6000 voxels into 20 parcels. However, this performance is sufficient since we only employ balanced partitioning in the case of small scale testing data sets or when parcels obtained on real data are too big.

#### 2.3.2. Functional parcellation

The main goal of functional parcellation is to provide homogeneous parcels with respect to hemodynamics. It is mainly motivated by the JDE procedure which assumes that the HRF shape is constant within one parcel. To provide such parcellation, results obtained from a GLM analysis, or any given task-specific functional maps are clustered using different available algorithms: K-means, Ward or spatially-constrained Ward as provided by scikit-learn^6^. To objectively choose an adequate number of parcels, theoretical information criteria have been investigated in Thyreau et al. (2006): converging evidence for Γ ≈ 400 at a spatial resolution of 3× 3× 3 mm^3^ has been shown for a whole brain analysis leading to typical parcel sizes around a few hundred voxels (≈ 2.7cm^3^). As the parcel size is not fixed, some big parcels may arise from the parcellation process and may slow down the overall parallel processing. To overcome this, the maximum parcel size was controlled by splitting too big parcels (larger than 1000 voxels) according to the balanced partitioning presented in section 2.3.1, which also guarantees the spatial connexity and thus properly satisfies the JDE assumptions on the HRF.

Such “hard clustering” approach yields sharp parcel boundaries that prevent from capturing smooth transitions between HRF territories. To avoid wrong boundaries, one can resort to over-segmented parcellations (high number of parcels).

## 3. pyhrf

The installation of pyhrf relies on the setuptools python package and requires the following dependencies: numpy^7^ and scipy^8^ for core algorithms, nibabel for nifti or gifti input/outputs, nipy for the GLM implementation and parcellation tools, matplotlib for plots and PyQT4 for GUIs. Optional dependencies comprise joblib, scikit-learn and soma-workflow. pyhrf is mainly intended for linux-based distributions as it has especially been developed under Ubuntu. Installation notes and documentation can be found online at http://www.pyhrf.org. Within the package, the following data files^9^ are shipped:

- two volume-based fMRI data sets (paradigm as CSV files, anatomical and BOLD data files). One serves quick testing while the other is intended for validation/demonstration purpose, which is used to generate results in section 4.3,
- one surface-based fMRI data set mainly intended for testing,
- several simulation resources in the form of png images to provide 2D maps of various activation labels and HRF territories.

The rest of this section is organized as follows. First, the overall workflow of how to use pyhrf is presented, which mainly resorts to command lines and some dedicated GUI tools. Second, to go further into the package architecture and also to address some features available when scripting, the design of pyhrf is introduced. Third, distributed computation is explained in terms of resource handling. Finally, the pyhrf viewer is presented with a focus on ergonomics.

### 3.1. Workflow

The typical usage of pyhrf relies on shell commands which work on XML files. This XML format was chosen for its hierarchical organization which suits well the nested nature of the algorithm parametrizations. A dedicated XML editor is provided with a PyQt4 graphical interface for a quick editing and also a better review of the treatment parameters. When such an XML setup file is generated, it defines a default analysis that involves a small volume-based real data set shipped with the package. This allows for a quick testing of the algorithms and is also used for demonstration purposes. Here is a typical example of shell commands used to perform a JDE analysis:

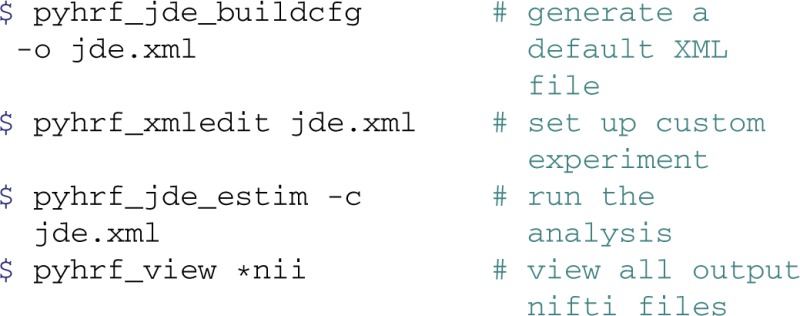



The “buildcfg” command offers various options to define setup items from the command line without having to edit the XML file. For example, the paradigm can be loaded from a CSV or a SPM.mat file. As for the JDE procedure specifically, the option --vem enables the variational EM approach developed in Chaari et al. (2013).

### 3.2. Design

**Figure 3:**
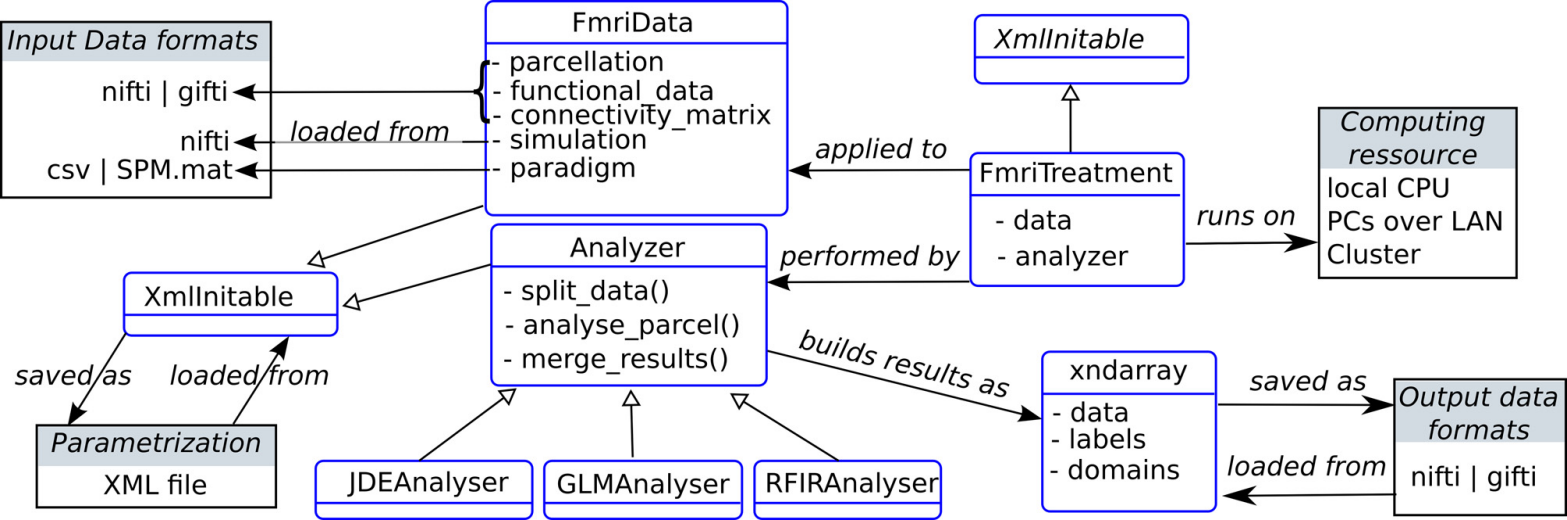
**Static organization of the main components in the pyhrf package (not exhaustive)**. Classes are represented as rounded blue rectangles and external resources (file, computing units) as black rectangles. Note that the XmlInitable class is duplicated for layout convenience. As in UML class diagrams, arrows have the following meaning: → stands for an association, 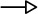 stands for a generalization.

An overview of the static design of the main package components of the package is shown in Figure 3. The class FmriData is the within-subject fMRI data representation, for any spatial support: on the cortical surface, in the volume, or from a simulation. This data representation comprises spatially flat data (fMRI time series and parcellation) and a connectivity matrix which holds the data topology. At the center of the analysis component is the Analyzer class that handles parcelwise data splitting which is done according to the input data parcellation by default, and also takes care of merging parcel-specific outputs at the end of the analysis. This Analyzer class is then specialized into various method-specific analyzers: GLM, RFIR and JDE. Note that the analyzer component is decoupled from the data component, as is classically done in scientific programming because they do not have the same life-cycles (e.g., the same model can be applied to various data objects). The FmriTreatment packs the data and analysis definitions together and handles distributed computation across parcels.

In the following sub-sections, two specific components are further explained: XML parametrization through the XmlInitable class, and the handling of arrays with axis semantics through the xndarray class.

#### 3.2.1. XML parametrization

**Figure 4:**
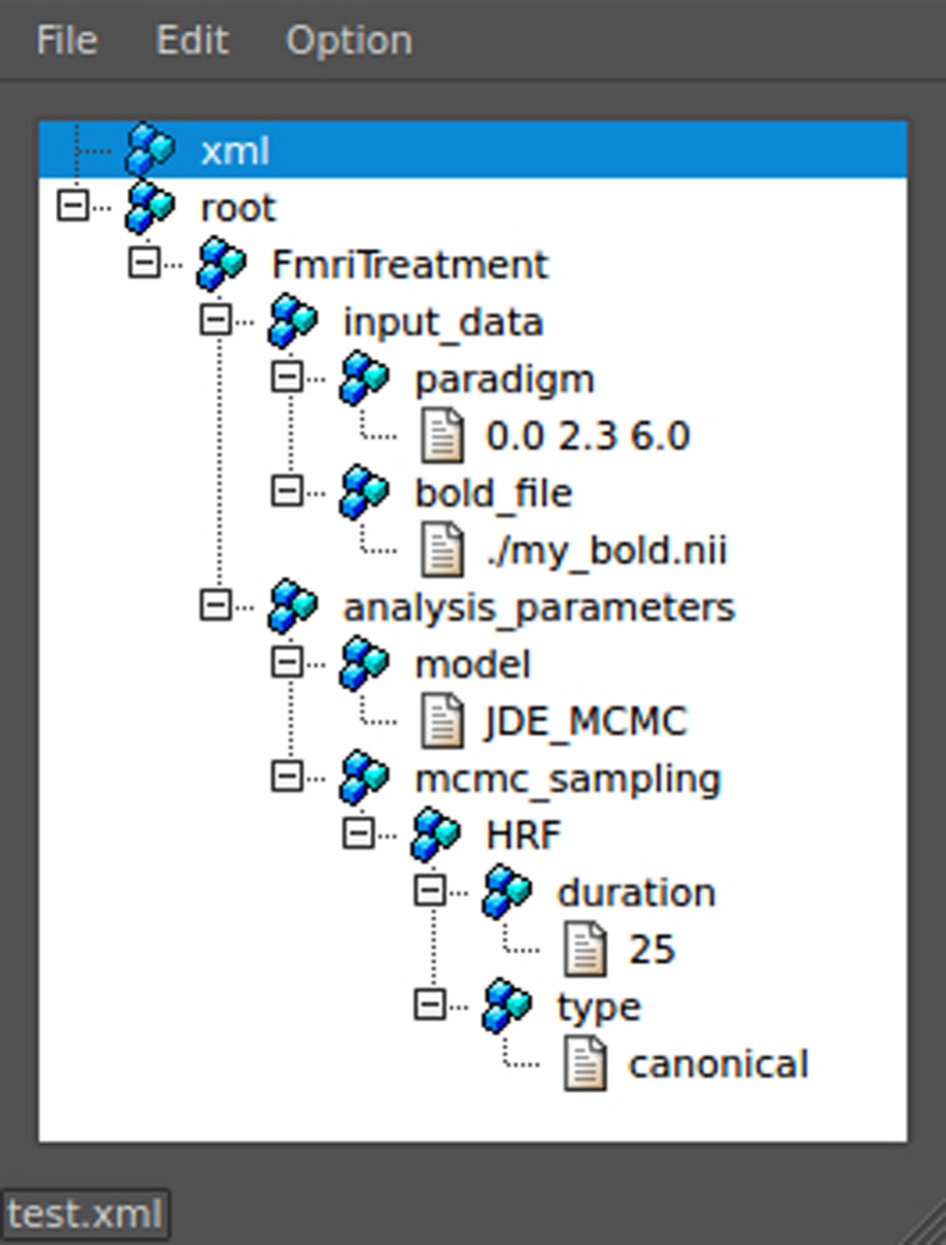
**Handling of XML parametrization**. The **top** part shows a code snippet that defines a dummy yet typical fMRI treatment structure with nested components. The init process of the resulting top-level object is then saved in an XML file. The **bottom** part is a snapshot of the pyhrf_xmledit main window where the XML file generated by the code snippet is browsed.

The XML format was chosen for its hierarchical organization which suits the nested nature of the algorithm parametrizations. Indeed, for a JDE analysis, here is an example of such different levels: treatment → analyzer → sampler → hrf sampler. At a given level, different classes may be used as there exist, for example, different sampler types depending on the type of prior expressed in the JDE model, so that we require a seamless parametrization process that avoids rewriting code for the building of parameter files each time a new model is tested. To do so, any object whose initialization has to be exposed in the XML configuration file inherits the XmlInitable class. This system is not a serialization process as the whole python object is not dumped in the XML. Only the parameters provided to the __init__ function are stored. In terms of object life cycle, this process handles object creation but is not able to track any subsequent modification. Figure 4 shows a python code sample that illustrates how the XML file is generated from this nested configuration situation. The resulting XML file as viewed by the command pyhrf_xmledit is also displayed.

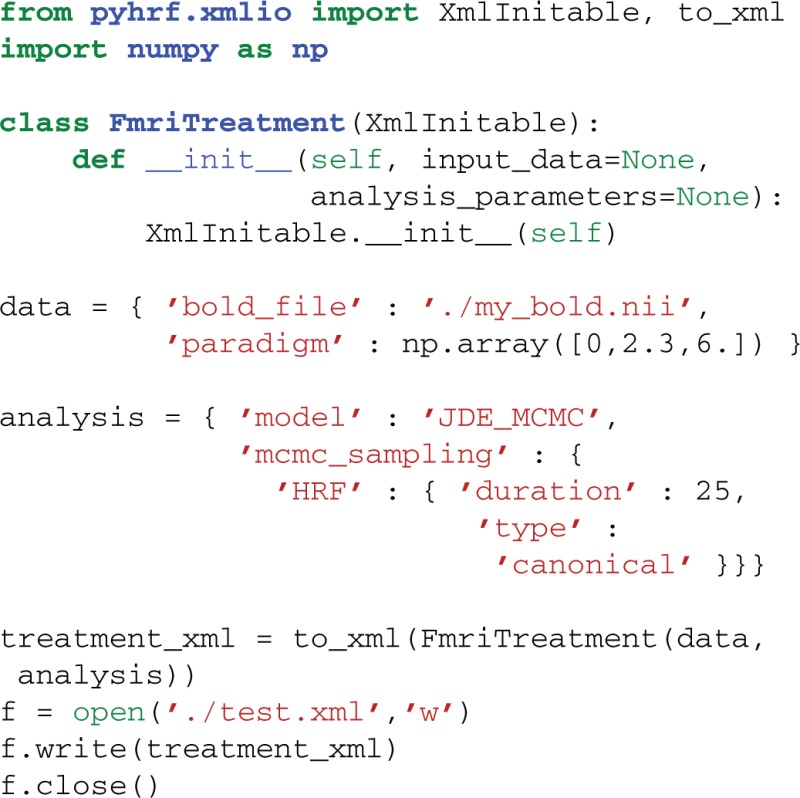



#### 3.2.2. The xndarray class: data array with axis semantics

The development of semantics-driven operations on data arrays was motivated by the parcel-driven nature of the analysis workflow which implied that parcel-specific results have to be merged in a transparent fashion, whatever their shape. Indeed, as pyhrf is the repository of all the methodological tools developed within the JDE framework, the number and the form of outputs is highly changing during the development and testing process. This involves producing convergence tracking, intermediate quantities in addition to the final results of interest. To avoid writing a specific saving procedure for such versatile and numerous outputs, the information about the interpretation of the data axes has to be explicit. The class xndarray handles any required reorientation prior to saving data arrays into nifti or gifti files. In the volume-based data case, the reorientation follows the nibabel convention that is sagittal, coronal, axial and time. To store the extra axis information along with the data, a dedicated nifti-extension is also written in the volume-based case or add a “pyhrf_xndarray_data” field in the gifti meta data dictionary in the surface-based case.

Moreover, outputs are primarily generated at the parcel-level so that they are in a flat shape, i.e., the position axis represent indices of positions in the spatial domain. To form the final whole brain outputs, the parcel-specific outputs have to be merged together and the position axis, if present, has to be mapped into the final spatial domain. Table 2 shows two examples of parcel-specific outputs that are merged to form whole brain data either by spatial mapping or by parcel stacking. To handle these two merging operations, stack and merge functions are provided. The reverse process is also available via the method explode which allows an array to be split according to a mask composed of integers, i.e., a parcellation. It returns the dictionary of “flat” parcel-specific data arrays associated with each integer label present in the mask.

**Table 2:** **Examples of merging operations performed on multiple parcel-specific data arrays, for some JDE outputs: parcel-specific HRFs and condition- and voxel-specific activation labels**.

	**Parcel-specific flat data**		**Merging operation**	**Whole brain data**	
	**Axis label**	**Axis domain**		**Axis label**	**Axis domain**
HRF	Time	[0, …, hrf_duration]	=		*same*
			∪	Parcel	[0, …, parcel_max]
Labels	Class	[’activ’, ’non_activ’]	=		*same*
	Condition	[’audio, ’video’]	=		*same*
	Position	[0, …, pos_max]	→	Axial	[0, …, axial_max]
				Coronal	[0, …, coronal_max]
				Sagittal	[0, …, sagittal_max]

*If the xndarray object contains the “position” axis, as for the “labels” object, then all parcel-specific results are merged into the same target volume and we depict the spatial mapping operation as “→” to map the “position” axis in to the spatial axes “axial,” “coronal,” and “sagittal.” For other axes aside from “position,” no merging operation is performed (“ = ” symbol). If the xndarray object does not contain the “position” axis, as for the HRF object, then all parcel-specific results are stacked and a new “parcel” axis is created (“∪” symbol)*.

In terms of data life cycle, xndarray objects are used to prepare data before analysis and to pack results after analysis. During the analysis process, it is more convenient to work with numpy arrays directly. The following code snippet illustrates the use of xndarray objects: functional and parcellation data are loaded, within-parcel means are computed and the result is saved to nifti:

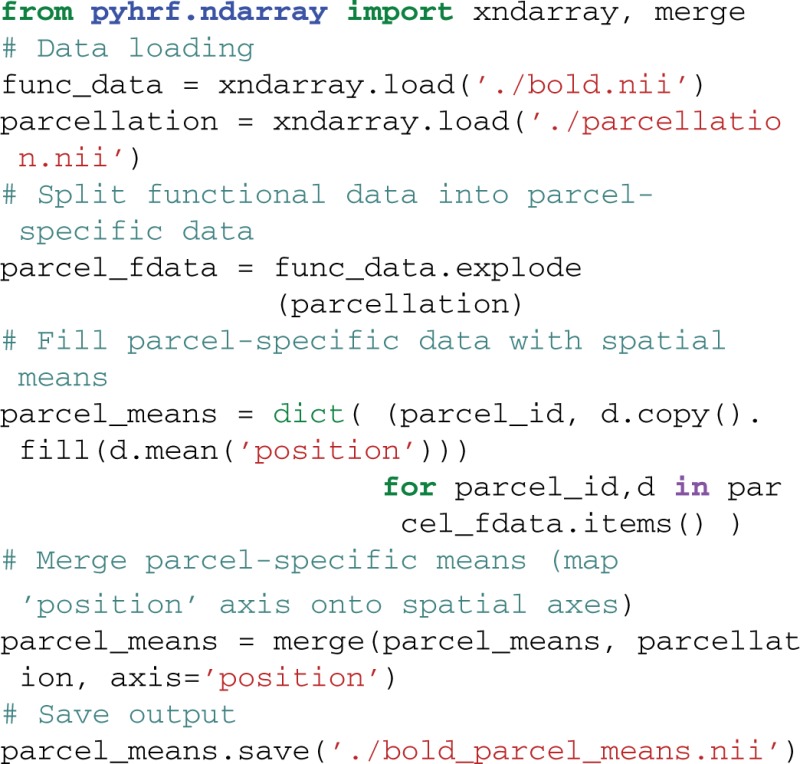



### 3.3. Distributed computing

PyHRF provides parallel processing features by exploiting local resources (multiple processors on a single workstation) as well as remote parallel processing units such as a local grid network or a cluster. A whole brain JDE analysis then boils down from 10 h to 15 min in parallel (on a 100-cores cluster). More precisely the available computing resources are handled as follows:

- **local multiple-cores CPUs:** through the use of joblib parallel features. The latter works by spawning python sub-processes that are then run on the different processing units by the operating system. The number of used CPUs can be setup by the user.
- **machines over a local area network:** through in-house code that relies on paramiko and hence uses *ssh* connections to distribute jobs on the LAN. A basic scheduler is implemented in pyhrf.grid that can also report faulty remote runs.
- **multi-core cluster:** through soma-workflow^10^ developed by Laguitton et al. (2011), which relies on paramiko^11^ on the client side and on DRMAA^12^ on the server side.

The distribution problem addressed here is a so-called embarrassingly parallel problem where the same treatment has to be repeated on several parcel-specific pieces of data. There is no shared memory management between distributed processes here.

To optimize the distribution process, the order in which the parcel-specific treatments are pushed in the process queue is done by pushing the biggest parcels first. To optimize also, a safeguard is imposed on the maximum parcel size (more than 7 cm^3^ in the volume or 11 cm^2^ on the surface). If a parcel exceeds this limit, it is divided up according to the balanced partitioning presented in section 2.3.1.2.

### 3.4. Viewer

**Figure 5:**
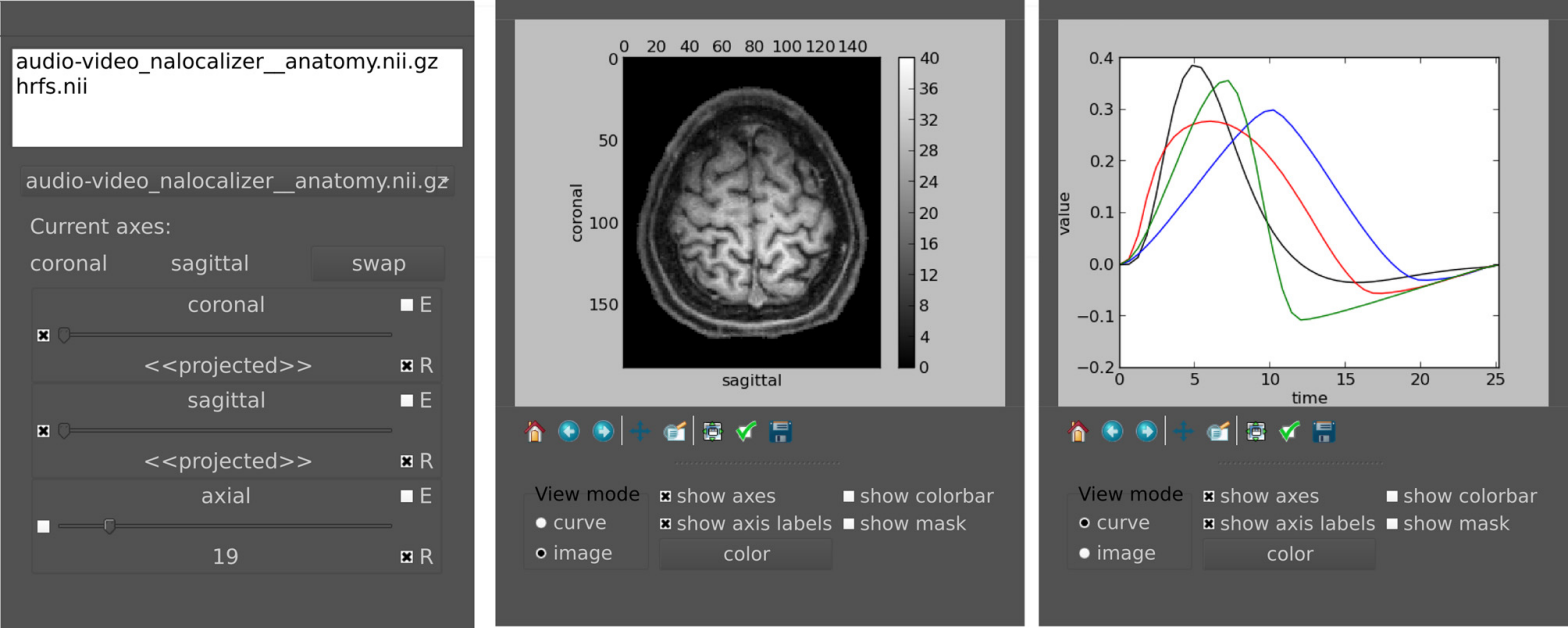
**Main widget components of pyhrf_view to browse and view *n*-dimensional data**. **Left:** The list widget on top displays the currently loaded objects. The slicer panel at the bottom allows: projection of axes (combo boxes on the left), domain value slicing (sliders in the middle) and definition of view synchronization (combo boxes on the right). For a given axis slicer, the two combo boxes defining synchronization are: **(E)** toggle emission of slice change to other slicers, **(R)** toggle reception from other slicers or from click events on plots. **Middle:** Plot window for the current selected slice. The top part displays the actual plot as produced by matplolib.pylab. The bottom part offers changing the view mode (either curve, image, or histogram), and toggling display of axes, colorbar and mask. The color button pops up a gradient map selector if in image mode or a color picker if in curve mode. **Right:** Other plot window to illustrate curve display.

pyhrf_view is a dedicated viewer built on PyQt4 which embeds a matplotlib view. The purpose of pyhrf_view is to provide convenient browsing of volume-based data^13^. However, it does not provide advanced overlaying features such as the display of functional over anatomical data. Instead, to plot the final “publication-ready” maps after having selected the results of interest with pyhrf_view, one can resort to the command pyhrf_plot_slice to directly generate a slice image of functional rendering along with anatomical overlay. One can also use a third party viewer such as Anatomist^14^, FSL_view^15^ or xjview^16^.

pyhrf_view offers *n*-dimensional browsing while most viewers in neuro-imaging software handle up to 4D volumes. In fact, there is a limit to the number of dimensions inherent to the nifti format which permits 7 axes at maximum. The viewer is composed of two main components (see Figure 5):

- a main window handling object and slice selection,
- plot windows which display the selected slice as curve or image.

The slice selection tools provides sliders to browse through axes domain values and display related information: axis name, current selected domain values and projection states. There can be up to two projected axes (2D), i.e., axes which will mapped to the actual plot axes. When multiple objects are loaded, slicers are synchronized to plotting views so that click events yield slider updates. This behavior can be modified in two ways. First, the reception combo box toggles whether the slider receives changes from other sliders. This is useful when one wants to prevent a given view from being updated by synchronization events (with reception off), e.g., when a reference slice should be compared to other slices. Second, the emission combo box toggles the spreading of slider changes to all other slicers. This is typically used to control a given axis across all displayed objects with a single slider (with emission on).

## 4. Results

### 4.1. Experimental paradigm

In all presented results, whether they focus on artificial or real data sets, we resorted to the same experimental paradigm. The latter is a multi-functional cognitive localizer paradigm designed in Pinel et al. (2007). This paradigm enables the mapping of cognitive brain functions such as reading, language comprehension and mental calculations as well as primary sensory-motor functions. It consists of a *fast event-related* design (60 stimuli, ISI = 3.75 s) comprising the following experimental conditions: auditory and visual sentences, auditory and visual calculations, left/right auditory and visual clicks, horizontal and vertical checkerboards.

### 4.2. Artificial data generator

Simulations in pyhrf mainly consist of building a script that defines a pipeline of versatile simulation bricks presented in Table 3. Writing a simulation script as a sequence of functions makes things difficult to read and to reuse. Instead, all simulation bricks are gathered inside a python dictionary that maps a simulation label to its corresponding value. This value can be directly defined as a python object or as a function which can depend on other simulation items and which is called when the simulation pipeline is evaluated. The pipeline structure arises from the link between simulation labels and function arguments. An example of such simulation script is given below:

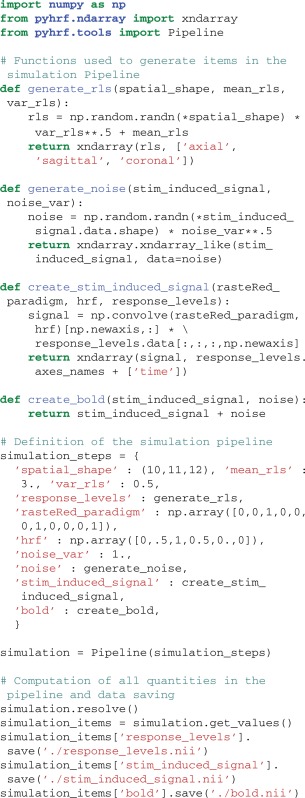



**Table 3:** **Different types of simulation bricks available in pyhrf**.

**Simulation item**	**Available generation process**
Experimental paradigm	Localizer, random event-related
Activation labels	Hand-drawn 2D maps, 3D Potts realizations
Response levels	Bi/tri mixture of Gaussian or Gamma components
Hemodynamic response function	Canonical, Bezier curve, Gaussian smooth
Low frequency drift	Polynomial, cosine
Noise	White, auto-regressive of order p

*The “localizer” paradigm is described in Pinel et al. (2007). Hand-drawn maps for activation labels are in the form of png images. Gaussian smooth generation of HRFs stands for the regularized prior used in the JDE model*.

**Figure 6:**
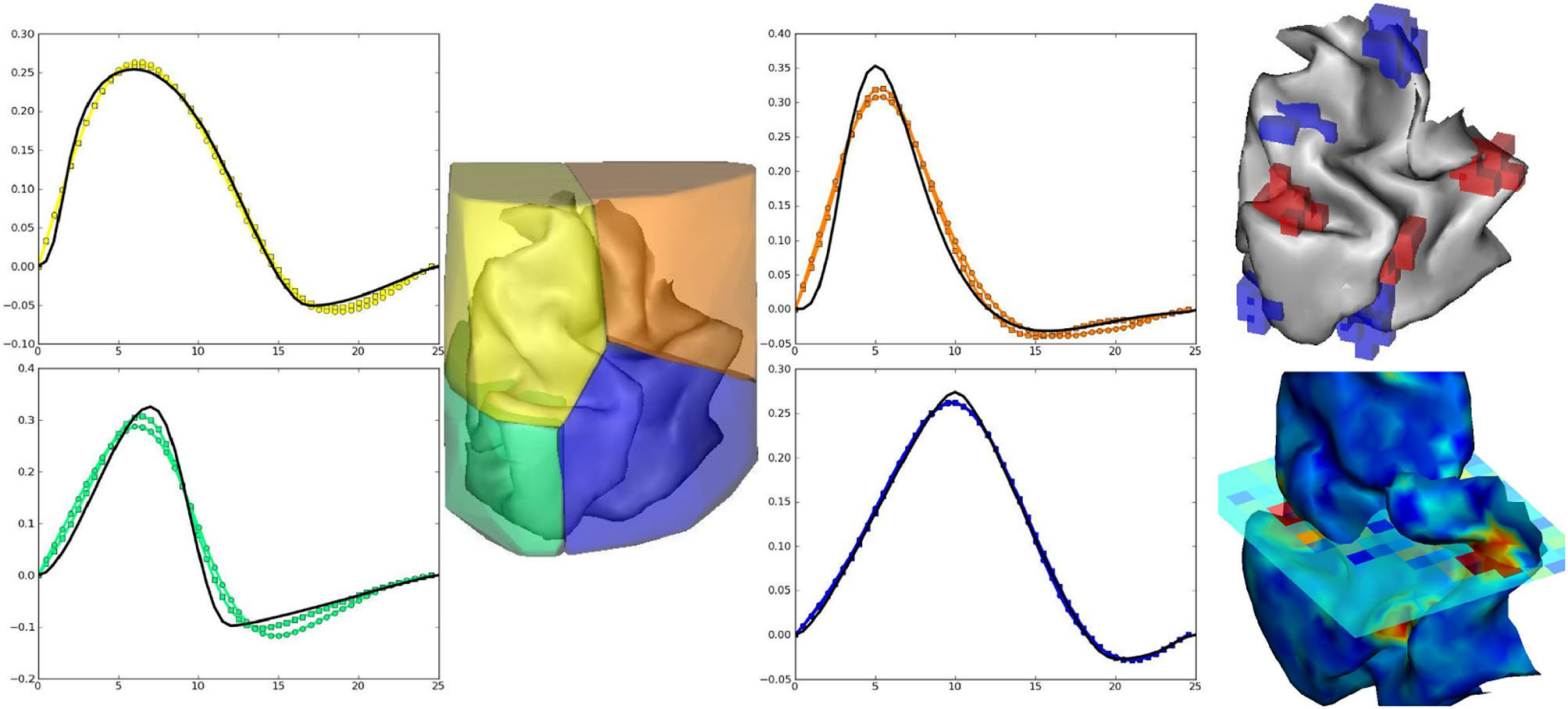
**Results on volume-based and surface-based artificial data**. **Left part**: HRF estimates obtained by JDE on the four artificial parcels. Ground truth HRFs are depicted in black line while colored HRF are HRF estimates that match the color of the parcels. **Right part, top**: labels simulated in the cortical fold for two conditions (in blue and red). **Right part, bottom**: Response levels estimates obtained by JDE on the cortical surface and in a selected slice of the volume. 3D renderings were produced with anatomist.

The artificial data experiment presented here comprises the generation of BOLD time series within the volume and then projected onto the cortical surface. To do so, shipped data defines a volume of 4 HRF territories, as well as the gray/white matter segmentation obtained from real data in the occipital region. Within the gray matter mask, activation labels are generated and conditionally to them, response levels are simulated according to a bi-Gaussian mixture. For the sake of simplicity, a version of the localizer paradigm presented in the previous section is merged over the auditory and visual modalities so as to obtain only two conditions. In all HRF territories this paradigm is then convolved with HRF generated by Bezier curves that enable the control of the time-to-peak and time-to-undershoot. Finally, nuisance signals are added (Gaussian noise and polynomial drift) to obtain the volume of artificial BOLD data. To generate surface-based data, data are projected onto a cortical fold that is also shipped in the package and we resorted to an external projection tool, developed in Operto et al. (2006) but others are available such as Freesurfer. Figure 6 presents the results obtained on artificial data using the JDE procedure. HRF estimates recover their respective ground truth profiles with a slightly more deformed curve obtained on the cortical surface for the bottom right (green) HRF territory, compared with the volume-based case. Detection results (response levels maps in Figure 6) also shows the correct recovery of the simulated ground-truth, in the volume and on the cortical surface.

### 4.3. Within-subject method comparison

The analyzed real data set, which is shipped with pyhrf, was a subset of an fMRI acquisition performed on a single healthy subject with a 3-Tesla Tim Trio Siemens scanner using an EPI sequence. The following settings were used for this acquisition: the fMRI session consisted of *N* = 128 scans, each of them being acquired using TR = 2400 ms, TE = 30 ms, slice thickness: 3 mm, FOV = 192 mm^2^ and spatial in-plane resolution of 2 × 2 mm^2^. In order to reduce disk usage and to focus only on areas of the brain which are expected to elicit activity in response to the paradigm, functional data was restricted to selected regions of interest that comprise occipital, temporal, parietal and motor regions. To improve interpretation and data plot rendering, an anatomical image is also shipped, with an in-plane resolution of 1 × 1 mm^2^ and slice thickness of 1.1 mm.

**Figure 7:**
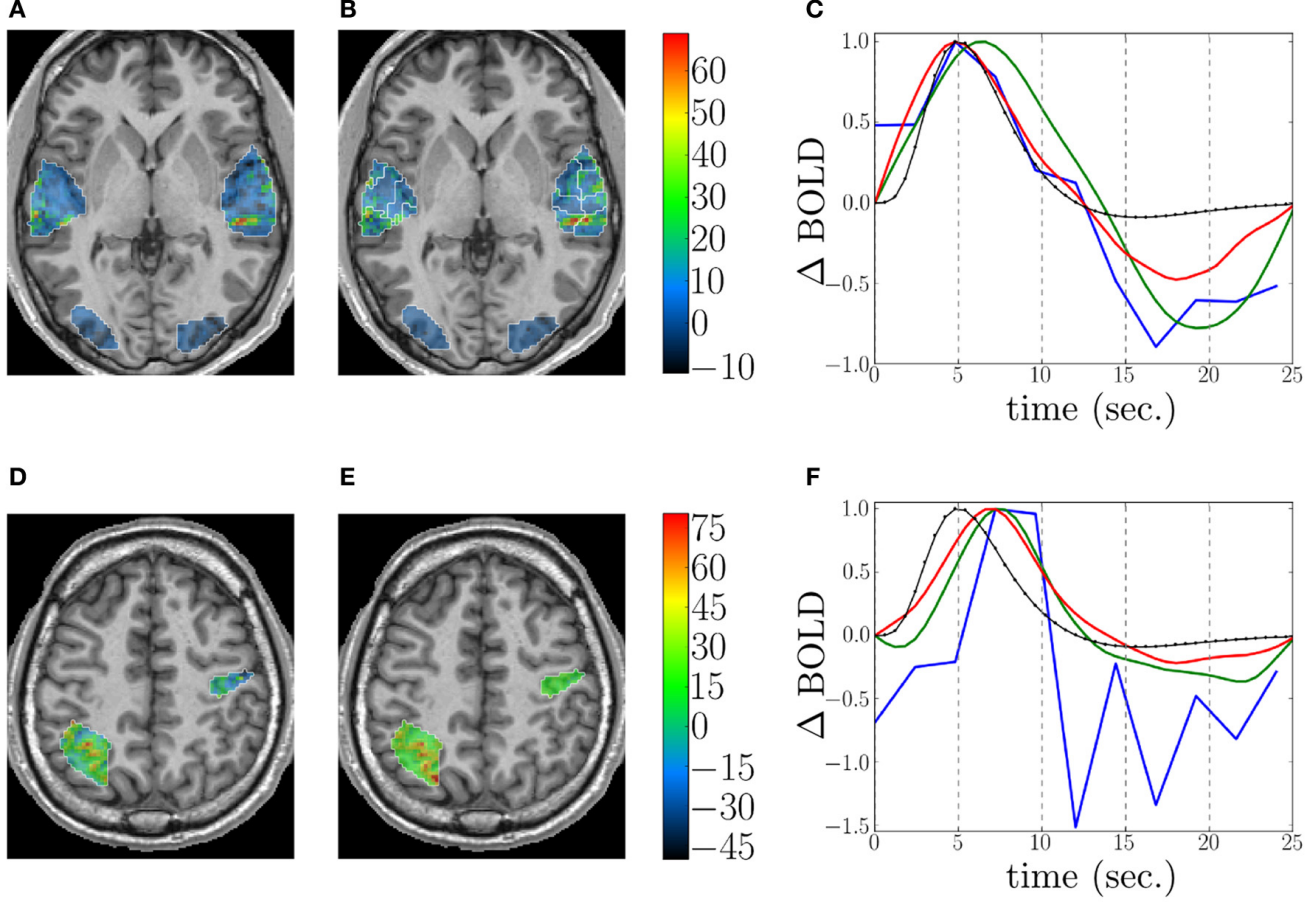
**Detection and estimation results on the shipped real data set**. Top and bottom rows: Auditory and computation experimental conditions, respectively. Columns from left to right: Response level maps, for **(A,D)** GLM with canonical HRF, **(B,E)** JDE, superimposed with the functional parcellation (white borders). Neurological convention: left is left. **(C,F):** Estimation results for GLM FIR (blue), RFIR (green), and JDE (red). The canonical HRF is shown in black.

This fMRI data set was analyzed using GLM with a canonical HRF, FIR, RFIR and JDE^17^. For JDE, the functional parcellation was built according to the method described in section 2.3.2. Figures 7A,B depicts detection results for the auditory effect, obtained by GLM with canonical HRF (see Figure 7A) and JDE (see Figure 7B). Both methods highlight the same activation localization, with a slightly stronger sensitivity for JDE. Figure 7C shows HRF estimation results as obtained by FIR, RFIR and JDE at the same local maximum on the left temporal region. Note that the HRF estimate provided by the JDE procedure is regional. The HRF profile delivered by FIR appears noisier than the JDE and RFIR counterparts. Also the temporal resolution of FIR is limited to the TR of input data. In contrast, RFIR and JDE offer an enhanced temporal resolution of 0.6 s In terms of timing, the FIR and JDE methods yield a peak at 5 s which is compatible with the canonical HRF that has been fitted on temporal auditory regions (Boynton et al., 1996). Accordingly, the HRF estimates obtained by RFIR seem over-smoothed. Overall, JDE enables reliable activation maps *and* HRF profiles that can roughly be obtained by separate GLM and FIR analyses. Figures 7D,E shows results on effect maps for the computation effect, obtained by GLM with canonical HRF (see Figure 7D) and JDE (see Figure 7E). JDE results have a higher sensitivity which can be explained by an estimated HRF that differs from the canonical version (see Figure 7F). More specifically on the HRF estimation results shown in Figure 7F, we can make the same comments as for the auditory results. However, the FIR HRF profile is here more chaotic and its peak is less easy to identify as the curve shows a plateau between 7 and 10 s

### 4.4. Group-level hemodynamics

**Figure 8:**
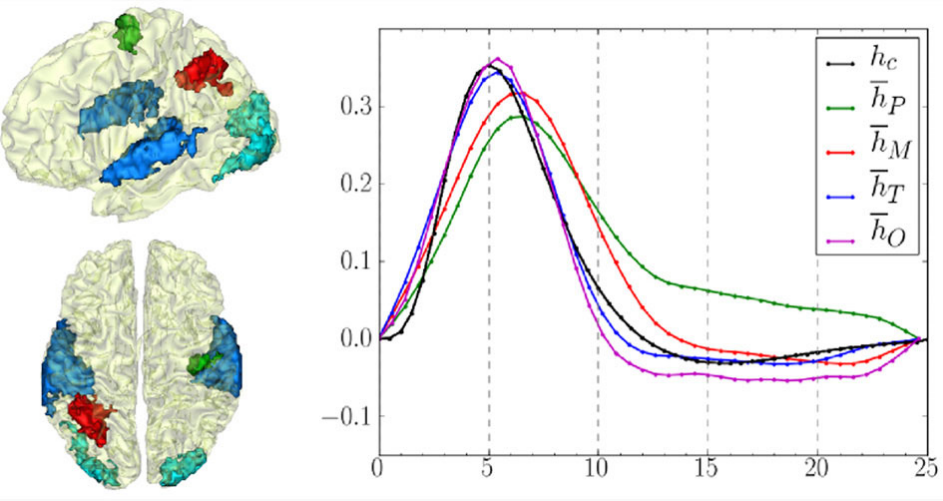
**Left:** Definition of regions of interest to investigate hemodynamics variability from JDE-based group-level analysis. **Top**: Sagittal view. **Bottom**: Axial/top view. Left parietal area (P) appears in 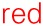, left motor area in the pre-central cortex is shown in 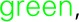 Bilateral temporal regions along auditory cortices and bilateral occipital regions in the visual cortices are shown in 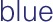 and 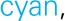 respectively. **Right**: Group-average HRF estimates for the four regions of interest: ***h***_*P*_, ***h***_*M*_, ***h***_*T*_, ***h***_*O*_ stand for HRF means in parietal, motor, temporal and occipital regions, respectively. ***h***_*c*_ correspond to the canonical HRF.

Using pyhrf, the hemodynamic variability was also studied on a group of 15 healthy volunteers (average: 23.2 years, std: 2 years). The experimental paradigm is described in section 4.1 and the fMRI acquisition parameters are similar to those previously mentioned in section 4.3. The results presented hereafter have been published in Badillo et al. (2013b). In this work, hemodynamic variability was investigated in four regions of interest, located in the left parietal cortex (*P*), bilateral temporal (*T*) and occipital (*O*) lobes and in the right motor cortex (*M*), as shown in Figure 8. These regions were defined after conducting a random-effect analysis to detect activation clusters showing a significant group-level effect. More precisely, we defined four contrasts of interest targeting brain activity in sensory and cognitive regions: a *Auditory vs. Visual* contrast for which we expect evoked activity in temporal regions in response, a *Visual vs. Auditory* contrast that induces evoked activity in the occipital cortex, a *Left vs. Right click* contrast for which we expect evoked activity in the right contralateral motor cortex, and a *Computation vs. Sentence* contrast which is expected to highlight activity in the frontal and parietal lobes specific to mental calculations. In terms of detection performance, at the group-level, JDE and GLM are comparable in primary sensory regions (where the canonical HRF is appropriate). However, in the parietal region involved in higher cognitive processes, the JDE approach yields more sensitive maps. In what follows, we summarize group-level hemodynamics results obtained in the regions of interest extracted from activated clusters.

The group-level HRF extraction in each ROI involves the following steps: For each subject, we identify the parcel containing the most activated voxel across stimulus-dependent response levels. Each individual parcel-based HRF time course is then scaled by the corresponding maximum response level so as to account for the inter-subject variability of the effect size. Last, each group-level HRF profile (see Figure 8) is computed as the average over the 15 subjects in the corresponding ROI.

One of the main results concerns the spatial gradient of discrepancy to the canonical HRF shape between regions. As shown in Figure 8, the mean HRF time courses retrieved in occipital and temporal regions are the closest to the canonical shape ***h***_*c*_. In the motor cortex, the HRF deviates a little more from the canonical filter, especially in terms of hemodynamic delay. Finally, the largest discrepancy to the canonical HRF was found in the parietal region.

## 5. Perspectives

### 5.1. Methodological perspectives

The main methodological developments are currently taking place in the JDE framework. In fMRI activation protocols, the paradigm usually consists of several runs repeating similar sequences of stimuli. For an increased stability of HRF estimates that cope with the between-run variability of the response magnitude, a hierarchical multi-run extension with heteroscedastic noise has been developed in Badillo et al. (2013c). It is particularly useful for pediatric imaging where runs are short in time. In the same vein of improving within-subject analyses, an approach to encode the condition-specificity at the parcel level is being developed to enforce non-relevant conditions to yield null activation, as in Bakhous et al. (2013).

The variational EM version of JDE that has been published in Chaari et al. (2013) and that appeared to be 10–30 times faster than its MCMC alternative, has allowed us to address (Chaari et al., 2012) the additional task of estimating the spatial aggregation support of HRF shapes (parcellation), which is supposed given *a priori* in the current JDE approach. The so-called joint Parcellation-Detection-Estimation (JPDE) validation is still ongoing. In an attempt to solve the same issue, an alternative based on random parcellations and consensus clustering has been recently proposed in Badillo et al. (2013a).

Closely related to the results presented in section 4.4, a multi-subject extension of the JDE is currently developed to properly account for the between-subject HRF variability and recover a meaningful and potentially less biased group-level HRF profile. This development trail will bring modification in the core design of pyhrf so as to take into account the new “group” data axis.

Finally, recent work has opened the path to multi-modality by processing Arterial Spin Labeling fMRI data (Vincent et al., 2013). To analyze such data, physiologically-inspired models are investigated to establish parsimonious and tractable versions of physiological models such as the balloon model described in Friston and Buechel (2000) and Buxton et al. (2004). Hence, for validation purpose, the artificial data generator is also being enriched with the simulation of physiological models.

### 5.2. Package perspectives

In addition to improving the documentation and usability of the current package version, additional developments will be first motivated by the above-mentioned methodological perspectives, namely re-factoring part of the data design to integrate the group-level and multi-session data components. This will mainly involve the modification of the FmriData class and the addition of a new FmriGroupData class. The handling of data input will have to be extended to exploit a hierarchy of subject-specific files.

We also plan to enrich the parcellation component by handling classical atlases such as the Automated Anatomical Labeling (AAL) atlas built by Tzourio-Mazoyer et al. (2002), the Brodmann regions (Brodmann, 1909) and the Harvard-Oxford atlas (Desikan et al., 2006) available in FSL^18^. The goal is to enable the definition of functional parcels that are consistent with previous studies in the literature and also to further investigate the anatomo-functional link by comparing atlas-driven vs. data-driven parcellations.

In order to offer more user-friendliness, the construction of a unified graphical user interface is foreseen, which will gather together the XML editor and the viewer while also enabling the selection of the analysis type. We also envisage resorting to wizard interfaces to guide the setup process and deliver contextual documentation. In terms of browsing features, tools to properly explore the surface-based results are currently missing, as we resort to an external tool, anatomist. The goal is not to reproduce all the features offered by the latter which enable the output of paper-ready figures through joint volume/surface rendering, data fusion and material handling. We rather think of a simple textured mesh viewer associated with a picking feature in order to synchronize other views. The main usage is to make the selection of a mesh node and the corresponding HRF estimate feasible. For making this surface-based rendering available, mayavi^19^ is an appealing candidate since it has been already intensively used in the python community.

Finally, we plan on incorporating GPU parallel computing features. This technology is becoming more and more available and powerful and may also appear cheaper than CPU computing systems (see Owens et al., 2008 for a review). Specifically, the NVIDIA chipsets are easily accessible for general purpose computing through the python package pyCUDA^20^. A simple test on matrix products with a complexity similar to that of our models showed a gain of one order of magnitude in favor of GPU computations^21^ (NVIDIA GeForce 435M graphics card) compared to CPU-based computations (Intel Core M480 @ 2.67 GHz) with numpy.

## 6. Conclusion

The pyhrf package provides tools to detect evoked brain activity and estimate the underlying dynamics from fMRI data in the context of event-related designs. Several “reference” methods are available: the GLM, FIR and RFIR approaches, and also more flexible models as provided by the JDE framework. The choice of the analysis tools depends on the experimenter's goal: if simple mappings are required, the GLM is appropriate provided that the HRF is expected to be close to its canonical version, but for finer dynamic estimation, the JDE procedure is more suitable. The design of pyhrf allows the handling of volume-based and surface-based data formats and also the utilization of several distributed computing resources. The main user interface is done by shell commands where the analysis setup is stored in an XML configuration file. Two graphical components are provided: an XML editor and a *n*-dimensional volume-based data browser.

This package provides valuable insights on the dynamics of the cognitive processes that are not available in classical software such as SPM or FSL. Hence, it offers interesting perspectives to understand the differences in the neuro-vascular coupling of different populations (infants, children, adults, patients, etc.).

### Conflict of interest statement

The authors declare that the research was conducted in the absence of any commercial or financial relationships that could be construed as a potential conflict of interest.

## Footnotes

^1^www.nipy.org

^2^www.nipy.org/nibabel

^3^Over-sampling could be performed in the case of FIR GLM but is not advisable in terms of estimability since some FIR coefficients may be poorly or even not associated with paradigm covariates in matrix ***X***^*m*^, depending on the paradigm jittering.

^4^Note that the RFIR approach with supervised regularization is much faster with an analysis duration of 20 min. since the maximum *a posteriori* estimator admits a closed form expression.

^5^implemented in scipy.spatial.KDTree

^6^sklearn.cluster.ward

^7^www.numpy.org

^8^www.scipy.org

^9^There is no special licence on the shipped data sets.

^10^http://brainvisa.info/soma-workflow/

^11^http://www.lag.net/paramiko/

^12^http://www.drmaa.org/

^13^Surface rendering is not available. Anatomist is recommended for such usage.

^14^http://brainvisa.info

^15^http://fsl.fmrib.ox.ac.uk/fsl/fslview/

^16^http://www.alivelearn.net/xjview8/

^17^analysis scripts are available at http://github.com/pyhrf/pyhrf/tree/master/script/frontiersBIM14/

^18^http://fsl.fmrib.ox.ac.uk/fsl/fslwiki/

^19^http://code.enthought.com/projects/mayavi/

^20^http://developer.nvidia.com/pycuda

^21^benchmark available at http://wiki.tiker.net/PyCuda/examples/DemoMetaMatrixmulCheetah
